# Enhancing Gluten Network Formation and Bread-Making Performance of Wheat Flour Using Wheat Bran Aqueous Extract

**DOI:** 10.3390/foods13101479

**Published:** 2024-05-10

**Authors:** Cheng Li, Gengjun Chen, Michael Tilley, Richard Chen, Mayra Perez-Fajardo, Xiaorong Wu, Yonghui Li

**Affiliations:** 1 Grain Science and Industry, Kansas State University, Manhattan, KS 66506, USA; 2 Center for Grain and Animal Health Research, US Department of Agriculture, Agricultural Research Service, Manhattan, KS 66502, USA

**Keywords:** wheat bran extract, dietary fiber, gluten, dough rheology, bread

## Abstract

Wheat bran possesses diverse nutritional and functional properties. In this study, wheat bran aqueous extract (WBE) was produced and thoroughly characterized as a functional ingredient and improver for bakery application. The WBE contained 50.3% total carbohydrate, 24.5% protein, 13.0% ash, 6.7% soluble fiber, 2.9% insoluble fiber, and 0.5% β-glucan. Notably, adding 7.5% WBE significantly increased the bread-specific volume to 4.84 cm^3^/g, compared with the control of 4.18 cm^3^/g. Adding WBE also resulted in a remarkable improvement in dough properties. The WBE-enriched dough showed increased peak, setback, breakdown, and final viscosities, along with higher storage and loss modulus. Scanning electron microscopy analysis further revealed that the WBE promoted the aggregation of protein and starch within the dough. The extractable gliadin to glutenin ratio increased with 5 and 7.5% WBE additions, compared with the control and 2.5% WBE addition. WBE did not significantly alter the starch gelatinization temperature or dough extension properties. These findings demonstrate that the inclusion of WBE in wheat flour is a promising approach for producing high-quality bread that is enriched with dietary fiber and protein.

## 1. Introduction

Bread, a widely consumed staple food, has a remarkable global consumption of approximately 70 kg per person annually, with annual production exceeding 9 billion kilograms [1]. Functional natural ingredients are highly demanded in bakery applications due to their eco-friendliness and clean-label nature, requiring further investigation [2]. Wheat bran, an underutilized byproduct from the wheat milling industry, is a valuable source of high-quality nutrients, including dietary fibers, proteins, vitamins, and bioactive compounds, and has attracted much interest in food development. It comprises the outer layers of the wheat grain and possesses a layered structure consisting of the outer pericarp (mainly composed of cellulose, hemicellulose, and minimal lignin), inner pericarp (rich in lignin), aleurone layer (abundant in hemicellulose and protein), and seed coat [3]. Wheat bran hemicellulose, primarily arabinoxylans, exhibits diverse physicochemical properties [4], making these components valuable for food applications, particularly in baking, to enhance economic returns in cereal processing.

Direct utilization of wheat bran in bakery products is limited due to its adverse effects on dough mixing properties and end-use characteristics, including rheological aspects, appearance, and textural and sensory attributes. Previous studies suggest that certain components in bran, such as water-soluble fibers and proteins, could increase loaf volume and impart a soft texture to bread crumbs [5,6]. However, researchers also found that adding water-insoluble arabinoxylan (2.5 to 7.5%) reduced dough elasticity and hindered protein formation, potentially resulting in undesirable bread quality, such as smaller loaf volume and excessive crumb hardness [7]. The presence of insoluble arabinoxylan also hinders the formation of disulfide bonds and thermal aggregation of gluten [8]. Despite previous research indicating the beneficial effects of soluble and insoluble arabinoxylan on gluten and dough properties, the existing extraction methods are often complex or expensive. Therefore, this study aims to extract wheat bran and develop simple and effective methods to enhance bread quality. The water-soluble components in wheat bran were extracted, and the monosaccharide composition in the wheat bran extract (WBE) was analyzed. The effects of WBE at various concentrations (0 to 7.5%) on surface morphology, gluten structure, dough rheology, and bread-baking quality were investigated. The findings of this study offer new insights into utilizing WBE as a bread enhancer.

## 2. Materials and Methods

### 2.1. Materials

Refined wheat flour (11.22% protein and 7.9% moisture content), food-graded malt, shortening, salt, sucrose, and yeast were purchased from the local market. Hard winter wheat bran was kindly provided by Hal Ross Flour Mill (Manhattan, KS, USA). All the chemicals and reagents used in this study were supplied by Fisher Scientific (Hampton, NH, USA) or Sigma-Aldrich (St. Louis, MO, USA).

### 2.2. Extraction and Characterization of the WBE

#### 2.2.1. Extraction of WBE

Wheat bran was mixed with distilled water at a ratio of 1:15 (*v*/*w*). After 2 h of stirring at room temperature, the mixture was centrifuged at 8000 g for 15 min. The supernatants were collected and frozen at −20 °C and then lyophilized. The resulting lyophilized powder, labeled as the WBE, was kept at −20 °C for future analysis.

#### 2.2.2. Proximate Composition Analysis

The protein, moisture, and ash of the WBE were determined using the approved AACC methods, 46-30.01, 44-15.02, and 08-03.01, respectively [9]. A nitrogen factor of 5.8 was used to calculate the protein content. Dietary fiber was determined according to AOAC 991.43 [10]. The crude fat content was estimated by extracting total lipids with ethyl ether. Total carbohydrate was obtained by subtracting the amounts of moisture, crude protein, crude lipid, and ash content from 100. The measurement of β-glucan was conducted according to AOAC 995.16 [11].

#### 2.2.3. GC–MS Monosaccharide Composition Analyses

To analyze the monosaccharide composition of WBE, a gas chromatograph (Varian CP-3800, Varian, Inc., Palo Alto, CA, USA), equipped with a CP-8400 autosampler and interfaced with a Varian 1200L triple Quadrupole MS/MS mass spectrometer (GC–MS), was employed, based on the procedure outlined previously [12]. In brief, the lyophilized WBE was hydrolyzed at 100 °C for 4 h using 2.5 M of trifluoroacetic acid (TFA). After the hydrolysate was dried using nitrogen gas, the sample was acetylated with acetic anhydride and pyridine. It was then incubated for 2.5 h at 100 °C. Next, sugar alditols obtained from the hydrolysate were dissolved in dichloromethane (DCM) and analyzed using the GC–MS. The analysis was performed by scanning (100 to 350 amu) at 2.14 scans per second, with the GS–MS transfer line maintained at 260 °C. The data were processed using Varian MS Workstation software 6.3 version. 

### 2.3. Mixograph Characteristics

The tested flour was prepared with refined flour with an additional four levels of lyophilized WBE (0, 2.5, 5, and 7.5 percent) for all the dough tests. The dough mixing characteristics were assessed in duplicate using a Mixograph (National Manufacturing, Lincoln, NE, USA) according to the AACC-approved method 54-40 [9]. The mixograms were processed using MixSmart software (v 3.40). After the flour (10 g, db) was mixed with the distilled water, the water absorption (%), dough development time (MPT, time to peak), peak value (PV), curve tail integral (CTI), breakdown, and slope were collected.

### 2.4. Pasting Characteristics

The pasting characteristics were evaluated with a Rapid Visco Analyzer (RVA) (PerkinElmer, Waltham, MA, USA). In this analysis, flour (3 g, 14% mb) was added to distilled water (25 mL). The mixture was heated, held at 95 °C, and cooled for a total duration of 13 min while being sheared at a constant shear rate of 160 rpm. The pasting temperature (TEMP), peak time, peak (PV), trough (trough), breakdown (BKD), setback (setback), and final viscosity (FV) were obtained. Each sample underwent triplicate analysis.

### 2.5. Dough Extensional and Dynamic Rheological Properties

The extensional and dynamic rheological properties of doughs were assessed using a TA-XTPlus texture analyzer along with the SMS/Kieffer rig (Stable Micro Systems, Godalming, UK) and Bohlin CVOR 150 rheometer (Malvern Instruments, Westborough, MA, USA) using the previous method [13]. Ten grams of the flour sample (14% mb) were used, and the mixing time was based on the Mixograph results. The shear storage modulus (G′) and loss modulus (G″) were evaluated as a strain of 0.01 to 10% at a frequency of 1 Hz. Each sample underwent at least triplicate analysis.

### 2.6. Scanning Electron Microscopy (SEM) Examination

The microstructure feature of the lyophilized WBE and lyophilized dough samples were viewed using an SEM (S-3500N, Hitachi Co., Tokyo, Japan) coupled with an energy-dispersive X-ray spectroscopy (EDS) detector with 10 mm^2^ Si (Li) at two magnifications (×100 and ×500). The system featured a working distance ranging from 11.6 to 12.3 mm and a voltage of 5 kV. The lyophilized dough samples were prepared by mixing refined flour and additional WBE (0–7.5 percent) in distilled water, with the optimized mixing time based on the Mixograph results, followed by lyophilization.

### 2.7. Thermal Properties

The lyophilized dough samples were subjected to thermal analysis via differential scanning calorimetry (DSC) (PerkinElmer, Claymont, DE, USA). Each sample (12 mg) and distilled water (36 mg) were weighed and scanned at a rate of 10 °C/min from 20 to 150 °C. To assess starch retrogradation, the sample was re-scanned after 7 days of storage in a refrigerator. Duplicate analyses were conducted for each sample. A reference was set using an empty DSC aluminum pan. The temperature and enthalpy values were recorded using PerkinElmer Diamond DSC Pyris software version 11.1.1, which was previously calibrated with pure indium.

### 2.8. RP-HPLC Analysis

The lyophilized dough sample (100 mg) was extracted twice with 1.0 mL of a salt solution (0.4 M of NaCl with 0.067 M of Na_2_HPO_4_/NaH_2_PO_4_ solution, pH 7.6) for 12 min at room temperature (albumin/globulin fraction); then, it was extracted twice for gliadin fraction using 1 mL of 60% ethanol for 12 min and extracted twice for glutenin fraction using 50% (*v*/*v*) propan-1-ol, 2 M of urea, 1% (*w*/*v*) dithioerythritol and 0.05 M of Tris-HCl for 45 min. The suspensions were centrifuged at 15,000× *g* for 10 min, and the same extracts were pooled and then filtered (Phenex filter membranes, Phenomenex, Torrance, CA, USA). The gliadin and glutenin fractions, and the ratio of gliadin to glutenin, were examined using HPLC (HP1050 Series, Agilent Technologies, Santa Clara, CA, USA) equipped with Agilent ChemStation software (Agilent, Santa Clara, CA, USA) following the previous method [13]. The gliadin and glutenin were identified at 210 nm, and the quantitative analysis relied on the measurement of peak areas obtained.

### 2.9. FTIR and Protein Secondary Structures

The PerkinElmer Spotlight 300 Spectrometer (PerkinElmer, Inc., Waltham, MA, USA) was used to measure the FTIR spectra of the lyophilized dough samples. Each sample was subjected to scanning within the range of 400–4000 cm^−1^, with air as the background, for a total of 64 scans. The spectra were converted to Gaussian shapes using OriginPro 2016 software to deconvolute the amide I region (1600–1700 cm^−1^).

### 2.10. Bread Characteristics

The bread was prepared following AACC method 10-10.03 [9]: 100 g of flour (14% mb), 3 g of shortening, 0.2 g of malt, 6 g of sugar, 1.5 g of salt, and 2 g of dry yeast. The mixing time and water addition were applied according to the Mixograph results. For each treatment, two loaves of bread were produced after 90 min of fermentation and 33 min of proofing. Baking was then carried out in a reel oven (National Manufacturing Co., Lincoln, NE, USA) at 215 °C for 24 min. After allowing the loaves to cool for 2 h, the weight was measured, and the volume was determined using AACC method 10-05.01 [9] The bread was subsequently cut into 15 mm thick slices. The cell properties of two middle bread slices were measured using a C-Cell Bread Imaging System (Calibre Control International Ltd., Warrington, UK) following AACC method 10-18.01 [9]. A TA-XT2 Texture Analyzer equipped with a 25 mm diameter cylinder probe (Stable Micro System, Godalming, UK) was applied to assess the texture characteristics of the slices at 1.0 mm/s testing speed, 50% strain, and 5 g trigger force. The color of the bread crumb was assessed using a digital precise colorimeter (Cielab, Guangzhou, China). The values of L* (white), a* (red), and b* (yellow) were measured, and three replicates were performed on the center slice.

### 2.11. Statistical Analysis

Statistical analysis was conducted using SAS Studio (SAS Institute Inc., Cary, NC, USA) employing one-way analysis of variance (ANOVA). All the assays were conducted on duplicate samples. Significance was determined at a *p*-value of less than 0.05 using Duncan’s test.

## 3. Results and Discussion

### 3.1. Chemical Composition of WBE

The WBE was obtained by water extraction with a yield of 15.7% (*w*/*w*), and the proximate chemical compositions of the WBE are shown in Table 1. The analysis reveals that WBE is rich in carbohydrates (50.3%) and protein (24.3%). Furthermore, the WBE contains 9.5% total dietary fiber and 4.35% crude fat. These findings align with the previous literature results regarding the chemical composition of a wheat bran aqueous extract [14]. A similar concentration of 22.7% protein in the extract highlighted the presence of Asp and Glu in considerable proportions regarding the amino acid profile [14]. The significant presence of acidic amino acids in albumins, which are more water-soluble, is expected due to the extraction method employed in this study. A high ash content (13%) in the extract was observed, which can be attributed to the co-extraction of minerals from the wheat bran. While the extraction process can potentially enhance the nutritional values in WBE by providing considerable minerals, the composition of the WBE exhibited heterogeneity, with proportions of protein, dietary fiber, and a significant portion consisting of components yet to be determined.

### 3.2. Sugar Composition and β-Glucans Analysis

The sugar composition analysis of the WBE revealed the presence of glucose, galactose, arabinose, xylose, and mannose at 7.09, 1.28, 0.57, 0.53, and 0.11%, respectively (Table 1). The concentration of β-glucans in the WBE was 0.52%. Previous studies have associated the incorporation of 0–0.75% yeast β-d-glucan in the dough with improved dough strength and bread quality [15]. The ratio of Ara (arabinose) to Xyl (xylose) (A/X) serves as an indicator of the degree of substitution of the xylan backbone with Ara residues, and the ratio varies depending on the anatomy of the wheat [14]. Previous research reported that A/X in the water-extractable arabinoxylans was 0.6, which was similar to the ratio in wheat bran [16]. According to Barron and et.al. [17], the outer pericarp layer had a higher level of degree substitution (average A/X of 1.16) than the aleurone and intermediate layers (average A/X of 0.44). The high ratio of A/X is 1.08 in the WBE, suggesting that it predominately originated from the outer pericarp of wheat bran. The discrepancy in A/X suggests the isolation of distinct layers within the wheat bran.

### 3.3. Morphologies of WBE and Dough

**Figure 1:**
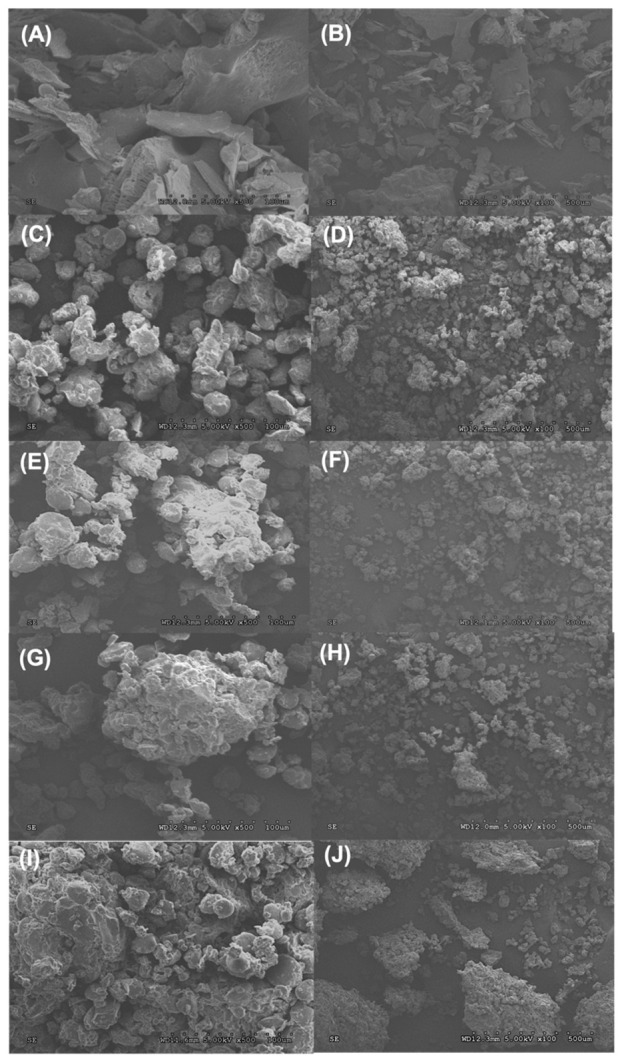
Microstructural analysis of wheat bran extract (WBE) and WBE-supplemented dough by scanning electron microscopy (SEM). WBE (**A**, ×500; **B**, ×100), control dough (**C**, ×500; **D**, ×100), 2.5% WBE dough (**E**, ×500; **F**, ×100); 5% WBE dough (**G**, ×500; **H**, ×100), 7.5% WBE dough (**I**, ×500; **J**, ×100). Control dough was made from white flour, 2.5–7.5% WBE dough, and dough at different WBE levels (2.5–7.5%).

The morphologies of the WBE and dough at different WBE levels were observed through SEM at a magnification of ×100 and ×500. The microstructure of the WBE is depicted in Figure 1A,B. Because most starch granules and adherent endosperm protein present in wheat bran are not soluble during extraction, WBE exhibits a laminated and sheet-like morphology, with edge folds on the surface composed of relatively rough flake-like elements. Previous research has reported a similar sheet-like form of polysaccharides extracted from wheat bran, which aligned with the morphologies observed in the WBE [18]. Figure 1C, D illustrates the microstructure of the control dough sample without the addition of WBE. Starch granules were interconnected and trapped within the gluten matrix, creating a dense and structured network. As the amount of WBE added to the dough increased from 2.5 to 7.5% (Figure 1E–J), the gluten matrix gradually became more continuous and aggregated, with more granules binding together. This observation was opposite to previous studies where wheat bran particles disturbed the development of the gluten network under SEM [19]. In contrast, the addition of WBE promoted the continuity of the gluten network in the dough, and, at higher concentrations, it expanded the structure of gluten and facilitated the connection of starch granules to the gluten matrix.

### 3.4. Mixograph Properties

The addition of WBE had significant and positive effects on the Mixograph properties of the dough. Table 2 presents the mixing parameters of doughs prepared with white flour and varying levels of WBE addition. When WBE was added to white flour dough at the same 59% water absorption as the control dough, the peak mixing time ranged from 5.0 to 5.6 min, similar to that of the control dough. The peak values increased significantly as the WBE concentration increased from 0 to 7.5%, indicating that the addition of WBE resulted in a stronger dough. Curve tail integral, which represents the overall area covered by the Mixograph, was significantly higher in the doughs with the WBE addition compared to the control dough. However, no significant differences were observed among the various levels of WBE addition. This finding provides evidence that WBE enhances the strength of the dough, requiring more energy to break down during mixing. During mixing, it is reasonable to assume that WBE may interact with gluten, leading to gluten aggregation. Similar to some hydrocolloids, WBE exhibits significant functionality, which depends on the composition and functional groups involved in the formation of hydrogen bonds with either gluten or water [5,6]. A similar result was previously reported that adding 1 and 2% wheat endospermic cell wall to the wheat dough improved the peak values and dough stability [20].

### 3.5. Pasting Properties

**Figure 2:**
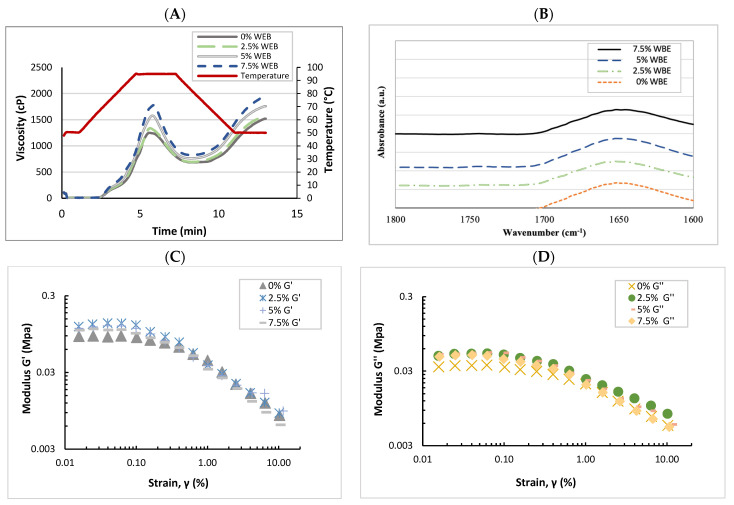
(**A**) RVA curves of white flour with the addition of WBE; (**B**) FTIR amide I bands (1600 to 1700 cm^–1^) of lyophilized dough; (**C**) Storage modulus G′ and (**D**) loss modulus G″ of the doughs (WBE) as a function of strain at 1 Hz. WBE, wheat bran extract; 0–7.5% WBE, dough made with white flour and 0–7.5% WBE addition.

The pasting parameters of the flour samples, as presented in Table 2 and Figure 2A, showed significant changes (*p* < 0.05) with the addition of WBE. The increasing concentration of WBE up to 7.5% resulted in a higher peak and final viscosity, ranging from 1239.0 to 1746.0 cP and 1508.0 to 1902.5 cP, respectively. Increased breakdown (577.0 to 932.0 cP) and setback (846.0 to 1088.5 cP) viscosities were also observed. Nevertheless, no significant differences were observed in peak time and pasting temperature among the treatments. The higher peak viscosity is likely attributed to the greater degree of starch granules swelling during heating. WBE may promote the interaction between swollen starch granules, leading to higher peak viscosity. A similar result was reported in a study on the effect of gum addition on chickpea–wheat flour, which suggested that increased peak viscosity may be due to increased intermolecular forces and chain entanglements within the starch paste [21]. The incorporation of WBE into the flour significantly increased the breakdown and final viscosities compared to the control (Figure 2A). This enhancement suggests that the mixture with WBE exhibits greater resistance to thermal degradation than the control flour [21].

### 3.6. Dough Extension and Dynamic Rheological Properties

The dough extension property offers insights into the viscoelastic behavior, which ultimately affects the quality of the end products. This is characterized by stress that surpasses the proportional increase in strain, which is closely correlated to the behavior of the dough [22]. According to the results presented in Table 2, the control dough had a rupture force of 15.27 g, while the doughs with added 2.5 to 7.5% WBE exhibited rupture forces ranging from 12.98 to 15.04 g. The variation in dough extension could be ascribed to the interaction facilitated by gluten cross-linking and aggregation influenced by the WBE.

The oscillatory rheological properties were assessed using a small deformation test through strain sweep, as shown in Figure 2C,D. The dough samples supplemented with WBE exhibited higher storage modulus (G′) and loss modulus (G″) than the control dough. A higher G′ indicates a more elastic material, thus dough containing a higher WBE concentration showed higher elastic properties. A previous study indicated that the viscoelastic characteristics of wheat doughs align with gels formed by reversible cross-linking, making them comparable to synthetic associative polymers [23]. The alternations in viscoelastic properties are probably a result of the interaction between WBE and gluten. Polysaccharide chains of the soluble dietary fiber in WBE, as the short-chain supplementation, could promote starch granule swelling and may also contribute to the increase in the G′ [24].

### 3.7. Thermal Properties

The gelatinization parameters of the flour samples, including peak height, onset, end, peak temperatures, and Delta H, are summarized in Table 2. Delta H represents the energy needed for the melting of a crystalline structure and can be used to analyze the degree of gelatinization of starch in cereal-based products [25]. No significant differences in the gelatinization values were found among the WBE samples and the control sample. This is likely due to the similar gelatinization temperature values [26], which is consistent with the result obtained from the RVA analysis. During the cooling stage, starch retrogradation occurs, leading to the re-crystallization of the amorphous structure in starch granules through hydrophobic interactions and hydrogen bonding. The addition of WBE to the flour samples had minimal impact on Delta H at 7 days since this effect was not statistically significant in terms of inhibiting starch retrogradation.

### 3.8. RP-HPLC of Gliadin and Glutenin Proteins

Wheat gluten primarily consists of two types of proteins: gliadin and glutenin. The quantitative data obtained from the RP-HPLC chromatograms of lyophilized dough samples with WBE are summarized in Table 2. At a low concentration (2.5%) of WBE, there was no significant change observed in the proportion of gliadin and glutenin fractions. However, at higher concentrations of WBE, a decrease in the proportion of glutenin proteins was observed. Specifically, the extractable glutenin decreased significantly from 40.93 µg/mg (control) to 27.76 µg/mg at 7.5% WBE. Thus, the decrease in glutenin extractability in the doughs might be due to the dilution of the glutenin protein in the system. The gliadin to glutenin ratio (gli/glu) has a crucial role in determining the dough’s maximum resistance and gluten index [27]. Gliadin contributes to the dough’s extensibility, while glutenin contributes to the dough’s elasticity and strength [28]. As shown in Table 2, as the level of WBE increases, the gli/glu also significantly increases from 1.06 to 1.59. This change in the gli/glu is likely to impact the physical and rheological properties of the dough.

### 3.9. FTIR Secondary Structure

The secondary structural pattern of gluten serves an important role in the formation of the gluten matrix. The α-helix and β-sheet secondary structures exhibit greater compactness and orderliness when compared to the random coil conformation, and the α-helix structure is relatively more stable than the β-sheet structure due to the presence of stronger hydrogen bonds [29]. FTIR spectra of gluten samples were collected to elucidate the effect of WBE on gluten conformation. The FTIR spectra reveal distinctive band regions within the amide I bands (1600 to 1800 cm^–1^), which provide indications of specific gluten secondary structures (Figure 2B). The amide I region was deconvoluted to determine the gluten secondary structures, and the peak assignments can be found in Table 2. The gluten structure of the dough was not significantly affected by the addition of 2.5 to 7.5% WBE (*p* > 0.05). Some previous reports clarified that the supplementation of dietary fiber or oat β-glucan increased ɑ-helix and random coil while decreasing β-turn and β-sheet structure [30,31]. The discrepancy may be due to the different extraction methods. The water-soluble fraction of wheat bran was used in our study, whereas earlier studies have utilized water-unextractable dietary fiber instead. In the starch-containing sample, the band at 1644 cm^−1^ is linked to hydrogen bonding, which is regulated by the presence of bound water in the non-crystalline portion of starch [32]. Identical intensities were observed at 1644 cm^−1^, indicating that the presence of the WBE in the samples had no significant impact on the hydrogen bonding within the starch hydroxyl group. This confirmed our results of the thermal properties measured by DSC.

### 3.10. Effect of WBE on Bread Quality

**Figure 3:**
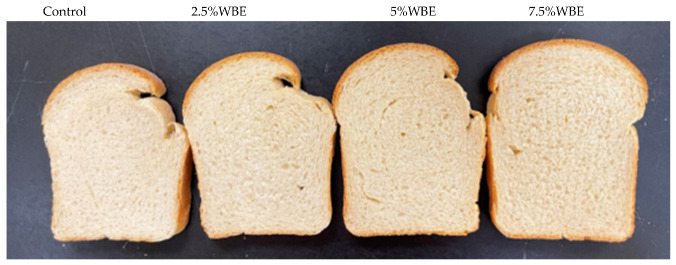
Photos of bread slices. Control: bread made from white wheat flour only; 2.5–7.5% WBE, bread made from white wheat flour, and an additional 2.5–7.5% wheat bran extract (WBE).

Bread-specific volume is known to be influenced by factors such as protein content and quality, as well as other grain and flour characteristics [33]. The specific volume (SV) of bread is a crucial characteristic that significantly influences consumer preference. Figure 3 presents the photos of bread slices, and the addition of WBE showed a significant facilitation effect on the SV (Table 3). The SV of bread with increasing WBE concentrations was significantly larger (4.30–4.84 cm^3^/g) compared to the control bread (4.18 cm^3^/g). The positive effect of the WBE can be mainly attributed to the interactions among fiber, gluten, and starch. A strong correlation between the increased peak/setback viscosity and increased bread-specific volume was found, which was also observed in the previous study [33,34] The increase in SV with the addition of WBE can be attributed to three factors. Firstly, WBE physically facilitates the coherence of the dough structure, as observed by SEM and RVA, leading to a larger SV. Secondly, the larger SV can be related to the enhanced cross-linking of gluten after adding WBE; the ratio of gli/glu may be partially responsible for the positive effects of the gluten complex on the bread characteristics. A previous study reported that a ratio of gli/glu of 1.82 resulted in a relatively higher bread-specific volume (3.61 cm^3^/g), whereas a ratio of 0.9 led to a lower volume (1.86 cm^3^/g) [35]. This result was in agreement with our finding, which indicated that WBE bread with a lower ratio of gli/glu has a s significantly higher specific volume than the untreated bread sample,. Moreover, soluble dietary fibers such as β-glucan have been observed to promote gluten aggregations within the gluten networks during the processes of dough mixing and heating [30,36].

The color properties of bread crumbs, as presented by the L*, a*, and b* values, are shown in Table 3. When 0 to 5% WBE was added to the bread formulation, no significant difference in color was observed compared to white bread. However, adding 7.5% WBE led to an increase in reddish and yellow tones, while the E value shows no significant difference compared to the control bread. The color of bread crust and crumb is primarily influenced by the Maillard reaction and caramelization processes that occur during baking. These reactions involve the interaction between amino acid residues and reducing sugars, resulting in the development of a brown color. The presence of distinct pigments and sugar in WBE could potentially explain the difference in bread color, with higher levels of WBE promoting these reactions and causing a deeper crumb color. Similar findings were reported in studies involving bran-enriched bread, where an increase in reddish and yellow tones was reported in whole wheat bread [37].

The WBE had an impact on the texture of the bread, resulting in similar or decreased values of bread hardness, gumminess, and chewiness compared to the control white bread, both on the same day and after 2 days of analysis (Table 3). In general, bread made with WBE had comparable or improved external traits, including soft and resilient crumbs, and a more uniformly distributed cell structure than white bread. The texture profile analysis (TPA) values for the white bread slices indicated the measurements for hardness, springiness, gumminess, and chewiness were 2.55 N, 168.59%, 2.12 N, and 3.57 N, respectively. In comparison, the bread with the addition of WBE showed similar hardness, springiness, gumminess, and decreased chewiness—in the range of 2.45–2.69 N, 131.06–154.33%, 1.87–2.01 N, and 2.63–2.92 N on the same day. After 2 days of storage, the bread with the WBE addition exhibited similar or lower hardness values, ranging from 6.95 to 7.75 N, compared to the control whose hardness was 8.16 N. A previous study revealed a positive impact of both water-extractable and unextractable pentosans on the bread crumb firmness, whereas water-soluble pentosans could promote the anti-staling effect [38]. The results of the C-cell analysis are presented in Table 3. The WBE bread had small and uniform cell structures, effectively retaining and utilizing the leavened gas within the gluten network throughout the process of fermentation and baking. Incorporating WBE promotes the gluten–starch network, which stabilizes the expansion of gas cells against disproportionation and coalescence, leading to an increased volume. Similar results were also reported when incorporating sodium alginate and xanthan gum in bread dough [39].

## 4. Conclusions

This study introduces a straightforward aqueous extraction method and highlights a significant positive influence of WBE on the microstructures of wheat flour dough and bread volume. Wheat bran extract (WBE) is rich in proteins, fibers, and minerals. WBE promotes the continuity of the gluten matrix and enhances the binding of flour particles. Incorporating WBE into refined flour also resulted in an increased gli/glu ratio and improved viscosity parameters, including peak, breakdown, setback, and final viscosities. The bread containing 7.5% WBE exhibited a remarkable 16% increase in specific volume compared to the control. Additionally, the bread with the WBE addition showed decreased chewiness and darker color compared to the control bread. WBE represents a promising and naturally sourced ingredient in bakery product formulation. Further research is suggested to investigate the key components within the extract and understand the mechanisms to better grasp its promising effects.

## Figures and Tables

**Table 1:** Proximate composition and sugar profile of wheat bran extract (WBE).

Proximate Analysis (%)		Dietary Fiber (%)		Sugar Profile (%)		β-Glucan (%)
Crude protein	24.25 ± 0.05	Soluble dietary fiber	6.7 ± 0.2	Xylose	0.53 ± 0.00	0.52 ± 0.01
Ash	13.0 ± 0.0	Insoluble dietary fiber	2.9 ± 0.2	Arabinose	0.57 ± 0.02	
Moisture	8.05 ± 0.05	Total dietary fiber	9.5 ± 0.1	Mannose	0.11 ± 0.00	
Crude lipid	4.35 ± 0.24			Glucose	7.09 ± 0.10	
Total carbohydrate *	50.3 ± 0.3			Galactose	1.28 ± 0.00	
				A/X	1.08 ± 0.03	

* The total carbohydrate content was estimated by subtracting the crude protein, crude fat, ash, and moisture content from 100. A/X means the ratio of arabinose to xylose.

**Table 2:** Physicochemical properties of doughs prepared from white flour with different levels of wheat bran extract (WBE).

*Mixograph*	Water Absorption (%)	Peak Time (min)	Peak Value (%)	Curve Tail Integral (%Tq × min)	Peak Width (%)	8 min Width (%)	
Control	59.0 ± 0.0 a	5.0 ± 0.0 a	36.4 ± 0.3 d	309.8 ± 5.3 b	20.3 ± 0.6 a	13.2 ± 0.2 a	
2.5%WBE	59.0 ± 0.0 a	5.3 ± 0.1 a	39.5 ± 0.1 c	334.1 ± 0.1 a	20.7 ± 0.4 a	11.1 ± 0.5 ab	
5%WBE	59.0 ± 0.0 a	5.6 ± 0.1 a	41.5 ± 0.1 b	342.6 ± 4.2 a	20.3 ± 0.2 a	10.4 ± 0.1 b	
7.5%WBE	59.0 ± 0.0 a	5.5 ± 0.1 a	43.0 ± 0.4 a	352.2 ± 2.4 a	30.2 ± 3.4 a	10.5 ± 0.5 b	
***RVA***	**Peak viscosity (cP)**	**Trough (cP)**	**Breakdown (cP)**	**Final viscosity (cP)**	**Setback (cP)**	**Peak time (min)**	**Pasting temperature (°C)**
Control	1239.0 ± 10.0 c	662.0 ± 24.0 b	577.0 ± 14.0 c	1508.0 ± 11.0 c	846.0 ± 13.0 c	5.5 ± 0.1 a	70.5 ± 0.3 a
2.5%WBE	1533.0 ± 11.0 b	796.0 ± 31.0 a	737.0 ± 20.0 b	1736.5 ± 21.5 b	940.5 ± 9.5 bc	5.7 ± 0.1 a	69.1 ± 0.4 a
5%WBE	1591.0 ± 15.0 b	770.0 ± 8.0 ab	821.0 ± 7.0 b	1770.5 ± 12.5 ab	1000.5 ± 4.5 ab	5.7 ± 0.1 a	69.4 ± 0.0 a
7.5%WBE	1746.0 ± 31.0 a	814.0 ± 12.0 a	932.0 ± 19.0 a	1902.5 ± 46.5 a	1088.5 ± 34.5 a	5.8 ± 0.0 a	69.0 ± 0.4 a
***Gelatinization***	**Onset (°C)**	**End (°C)**	**Peak (°C)**	**Peak height (mJ/s)**	**ΔH (J/g)**	**Dough extensibility Force (g)**	**Dough extensibility Distance (mm)**
Control	60.16 ± 0.45 a	71.32 ± 0.19 a	65.96 ± 0.68 a	0.28 ± 0.05 a	1.02 ± 0.21 a	15.27 ± 0.87 a	21.35 ± 3.53 a
2.5%WBE	58.49 ± 0.27 a	71.77 ± 0.33 a	65.19 ± 0.23 a	0.37 ± 0.00 a	1.57 ± 0.00 a	15.04 ± 2.01 a	29.98 ± 7.62 a
5%WBE	59.47 ± 0.18 a	71.94 ± 0.16 a	65.68 ± 0.05 a	0.38 ± 0.00 a	1.56 ± 0.01 a	12.98 ± 1.37 a	28.58 ± 5.82 a
7.5%WBE	59.56 ± 0.34 a	72.18 ± 0.01 a	65.78 ± 0.05 a	0.35 ± 0.02 a	1.44 ± 0.01 a	14.62 ± 2.91 a	30.97 ± 7.65 a
***Retrogradation***	**Onset (°C)**	**End (°C)**	**Peak (°C)**	**Peak height (mJ/s)**	**ΔH (J/g)**		
Control	32.03 ± 0.27 a	50.73 ± 1.07 a	42.24 ± 2.05 a	0.05 ± 0.01 a	0.33 ± 0.09 a		
2.5%WBE	31.70 ± 0.02 a	57.47 ± 0.81 a	43.86 ± 0.11 a	0.08 ± 0.02 a	0.87 ± 0.35 a		
5%WBE	32.17 ± 0.35 a	53.27 ± 0.52 a	42.56 ± 0.44 a	0.05 ± 0.00 a	0.36 ± 0.05 a		
7.5%WBE	31.77 ± 0.36 a	55.73 ± 2.60 a	43.75 ± 0.65 a	0.06 ± 0.00 a	0.47 ± 0.06 a		
***Chemical properties***	**Gliadin (µg/mg)**	**Glutenin (µg/mg)**	**Gli/glu**	**α-Helix (%)**	**β-Sheet (%)**	**β-Turn (%)**	**Random coil (%)**
Control	43.20 ± 0.53 a	40.93 ± 2.97 a	1.06 ± 0.06 b	40 ± 6 a	20 ± 4 a	6 ± 0 a	34 ± 4 a
2.5%WBE	41.12 ± 0.35 a	41.99 ± 0.36 a	0.98 ± 0.01 b	53 ± 8 a	18 ± 5 a	4 ± 2 a	25 ± 3 a
5%WBE	49.59 ± 5.21 a	31.10 ± 0.93 b	1.59 ± 0.14 a	45 ± 3 a	17 ± 3 a	6 ± 1 a	32 ± 6 a
7.5%WBE	43.09 ± 1.40 a	27.76 ± 0.41 b	1.55 ± 0.06 a	43 ± 3 a	15 ± 5 a	5 ± 1 a	37 ± 5 a

WBE, wheat bran extract; Control, white flour dough; 2.5–7.5% WBE, 2.5–7.5% WBE-addition dough. RVA, Rapid Visco Analyzer. Samples with different letters in the same column differ significantly (*p* < 0.05).

**Table 3:** Specific volume, crust color, C-cell properties, and texture profile analysis (TPA) data of bread with the addition of wheat bran extract (WBE).

Bread Type	Control	2.5% WBE	5% WBE	7.5% WBE
Specific volume (cm^3^/g)	4.18 ± 0.00 c	4.30 ± 0.02 c	4.52 ± 0.05 b	4.84 ± 0.02 a
L*	65.57 ± 0.13 a	66.37 ± 0.68 a	64.64 ± 0.75 a	64.72 ± 0.91a
a*	1.52 ± 0.06 b	1.56 ± 0.04 b	1.73 ± 0.08 b	2.08 ± 0.10 a
b*	16.31 ± 0.06 b	16.45 ± 0.48 b	17.39 ± 0.44 b	18.69 ± 0.34 a
E	67.59 ± 0.12 a	68.39 ± 0.77 a	66.96 ± 0.75 a	67.40 ± 0.88 a
Number of cells/areas (mm^2^)	0.66 ± 0.05 a	0.71 ± 0.06 a	0.67 ± 0.10 a	0.65 ± 0.04 a
Area of cells (%)	49.53 ± 0.68 a	49.73 ± 0.57 a	49.88 ± 0.62 a	50.30 ± 0.84 a
Wall thickness (mm)	0.45 ± 0.02 a	0.43 ± 0.02 a	0.45 ± 0.03 a	0.45 ± 0.01 a
Cell diameter (mm)	1.88 ± 0.06 a	1.82 ± 0.19 a	1.89 ± 0.22 a	1.91 ± 0.06 a
TPA on the same day				
Hardness (N)	2.55 ± 0.01 a	2.69 ± 0.17 a	2.45 ± 0.27 a	2.45 ± 0.15 a
Springiness (%)	168.59 ± 2.13 a	131.06 ± 24.53 a	150.15 ± 17.19 a	154.33 ± 10.08 a
Gumminess (N)	2.12 ± 0.08 a	2.01 ± 0.09 a	1.87 ± 0.17 a	1.90 ± 0.12 a
Chewiness (N)	3.57 ± 0.10 a	2.63 ± 0.48 b	2.84 ± 0.54 ab	2.92 ± 0.19 ab
TPA after two days				
Hardness (N)	8.16 ± 0.48 a	7.01 ± 0.64 b	6.95 ± 0.27 b	7.75 ± 0.16 ab
Springiness (%)	111.84 ± 20.28 a	116.64 ± 19.35 a	97.46 ± 2.73 a	101.38 ± 6.76 a
Gumminess (N)	4.74 ± 0.19 a	4.22 ± 0.54 ab	3.90 ± 0.37 b	4.38 ± 0.11 ab
Chewiness (N)	5.27 ± 0.72 a	4.93 ± 1.06 a	3.81 ± 0.46 a	4.44 ± 0.28 a

WBE, wheat bran extract; 2.5–7.5% WBE, bread slices made from control flour with 2.5–7.5% WBE addition. Values in the same row with different letters differ significantly (*p* < 0.05).

## Data Availability

The original contributions presented in the study are included in the article, further inquiries can be directed to the corresponding author.
